# Biodistribution of a Radiolabeled Antibody in Mice as an Approach to Evaluating Antibody Pharmacokinetics

**DOI:** 10.3390/pharmaceutics10040262

**Published:** 2018-12-05

**Authors:** Kevin J. H. Allen, Rubin Jiao, Mackenzie E. Malo, Connor Frank, Ekaterina Dadachova

**Affiliations:** College of Pharmacy and Nutrition, University of Saskatchewan, Saskatoon, SK S7N 5E5, Canada; kja782@mail.usask.ca (K.J.H.A.); jiaorubin9712@hotmail.com (R.J.); mem510@mail.usask.ca (M.E.M.); csf876@mail.usask.ca (C.F.)

**Keywords:** pharmacokinetics, antibodies, radiolabeling, biodistribution, mouse models

## Abstract

(1) Background: Monoclonal antibodies are used in the treatment of multiple conditions including cancer, autoimmune disorders, and infectious diseases. One of the initial steps in the selection of an antibody candidate for further pre-clinical development is determining its pharmacokinetics in small animal models. The use of mass spectrometry and other techniques to determine the fate of these antibodies is laborious and expensive. Here we describe a straightforward and highly reproducible methodology for utilizing radiolabeled antibodies for pharmacokinetics studies. (2) Methods: Commercially available bifunctional linker CHXA” and ^111^Indium radionuclide were used. A melanin-specific chimeric antibody A1 and an isotype matching irrelevant control A2 were conjugated with the CHXA”, and then radiolabeled with ^111^In. The biodistribution was performed at 4 and 24 h time points in melanoma tumor-bearing and healthy C57BL/6 female mice. (3) The biodistribution of the melanin-binding antibody showed the significant uptake in the tumor, which increased with time, and very low uptake in healthy melanin-containing tissues such as the retina of the eye and melanized skin. This biodistribution pattern in healthy tissues was very close to that of the isotype matching control antibody. (4) Conclusions: The biodistribution experiment allows us to assess the pharmacokinetics of both antibodies side by side and to make a conclusion regarding the suitability of specific antibodies for further development.

## 1. Introduction

The field of immunotherapy is experiencing explosive growth, with new antibodies being approved for clinical use, or being introduced into the research pipeline on a regular basis [1,2,3]. Monoclonal antibodies find applications in the treatment of multiple conditions, including cancer, autoimmune disorders, and infectious diseases. One of the initial steps in the selection of an antibody candidate for further pre-clinical development is determining its pharmacokinetics (PK) in small animal models. Usually PK studies are performed by administering the antibody candidate to the healthy mice, or a mouse model of a relevant disease, followed by harvesting organs and tissues at pre-determined time points. These samples are then digested and subjected to various downstream analytical techniques, such as mass spectrometry and immune-PCR (Polymerase Chain Reaction), in order to test for the presence of the candidate antibody [4,5]. These techniques are laborious and expensive and require access to state-of-the-art equipment, such as MALDI (Matrix Assisted Laser Desorption Ionization) mass spectrometers, as well as highly trained personnel for interpretation of the results. An alternative technique is to attach a radiolabel to the antibody of interest before administering it to mice, and then to follow its fate in vivo by measuring the amount of radioactivity present in the mouse organs and tissues at the pre-determined time points. Here we describe a straightforward and highly reproducible method for radiolabeling antibodies using commercially available linker and radionuclide, and performing biodistribution in a murine melanoma model.

## 2. Materials and Methods

### 2.1. Reagents, Antibodies, Radionuclides, and Cell Lines

The antibody to melanin, Ab1, was produced in our laboratories and human IgG isotype control Ab, referred to as Ab2 in the text, was purchased (Creative Diagnostics, Shirley, NY, USA). ^111^Indium was purchased as ^111^InCl_3_ from Nordion (Vancouver, BC, Canada). Bifunctional CHXA” ligand was purchased from Macrocyclics (Plano, TX, USA). Murine melanoma cell line B16-F10 was purchased from ATCC (Manassas, VA, USA).

### 2.2. Metal-Free Buffer Preparation

Stock buffers must be prepared as metal-free solutions in order to ensure contaminating metals do not interfere with downstream radiolabeling steps. All buffers were prepared as concentrated stocks with distilled/deionized H_2_O (ddH_2_O), using components purchased from Fisher Scientific (Ottawa, ON, Canada). Stock buffers were run through a Chelex cation exchange resin column to scavenge contaminating free metal ions.

The Chelex column was prepared by placing a glass wool plug in a glass chromatography column. The wool plug was rinsed with concentration HCl, followed by water until the eluate was a neutral pH. A slurry of Chelex-100, Na+ form, 200–400 mesh (BioRad, Hercules, CA, USA) was prepared in ddH_2_O and poured into the column in order to have approximately 5 cm of packed resin. The Chelex column was washed with ddH_2_O until the eluate returned to a neutral pH.

Conjugation buffer stock was prepared as 0.5 M Carbonate/Bicarbonate (0.02 M/0.48 M), 1.5 M NaCl solution at pH 8.6–8.7 in ddH_2_O. A prepared Chelex column was equilibrated with 100 mL of 10× stock buffer and the eluate was discarded. The remaining 10× stock buffer was run through the column and collected as a metal-free 10× stock. Conjugation buffer was prepared by diluting the 10× conjugation buffer by 10 with ddH_2_O and adding EDTA to 5 mM.

Ammonium acetate buffer was prepared as a concentrated stock (5 M, pH 7.5) and run through a Chelex column to scavenge free metal ions. Prior to completing Ab labeling it was diluted with ddH_2_O and used at 0.15 M concentration.

### 2.3. Radiolabeling of Antibody-CHXA” Conjugate with ^111^Indium (^111^In)

To conjugate the bifunctional chelator CHXA” to the antibody, the Ab must first be exchanged out of the storage buffer and into conjugation buffer. This was achieved by loading the Ab onto a 0.5 mL 30K molecular weight cutoff Amicon microconcentrator (Millipore, Burlington, MA, USA) with conjugation buffer and then centrifuging at 4 °C following the microconcentrator manufacturer’s recommended conditions. Conjugation buffer was added and centrifugation was repeated at least 10 times to ensure complete exchange of the Ab storage buffer to conjugation buffer. A 5-fold molar excess of CHXA” (2 mg/mL in conjugation buffer, prepared immediately before use) was added to the antibody solution. The reaction mixture was then incubated at 37 °C for 1.5 h. Upon completion of the reaction the Ab-CHXA” conjugate was then exchanged into the 0.15 M ammonium acetate buffer at 4 °C, the same as described for the exchange into the conjugation buffer. Protein concentration was determined by Bradford assay (BioRad, Hercules, CA, USA) prior to labeling the Ab.

The radiolabeling of the antibody-CHXA” conjugate with ^111^In was performed to achieve a specific activity of approximately 5 µCi/µg of the antibody. The amount of the radiolabeled antibody to be administered to a mouse was approximately 6 µg, therefore, the amount of radioactivity was 30 µCi. ^111^In chloride was diluted with 0.15 M ammonium acetate buffer and added to a microcentrifuge tube (MCT) containing the Ab-CHXA” conjugate in the 0.15 M ammonium acetate buffer, a minimum volume was desired, with a typical reaction volume being ~30 µL. The reaction mixture was incubated for 60 min at 37 °C. The reaction was quenched by the addition of 3 µL of 0.05 M EDTA solution to bind any free ^111^In.

The percentage of radiolabeling (radiolabeling yield) was measured by instant thin layer chromatography (iTLC) by developing 10 cm silica gel strips (Agilent Technologies, Santa Clara, CA, USA) in 0.15 M ammonium acetate buffer. In this system the radiolabeled antibodies stay at the point of application while free ^111^In, in the form of EDTA complexes, move with the solvent front. The strips were cut in half and each half was counted on a 2470 Wizard2 Gamma counter (Perkin Elmer, Waltham, MA, USA) that was calibrated for the ^111^In emission spectrum and only emissions in this range were considered in the counts per minute (CPM). The percentage of radiolabeling was calculated by dividing the CPM at the bottom of the strip (labeled antibody) by the sum of the CPM at the bottom and the top of the strip (total amount of radioactivity) and multiplying the result by 100.

### 2.4. B16-F10 Melanoma Tumor Model

All animal studies were approved by the Animal Research Ethics Board of the University of Saskatchewan (Animal Use Permit #20180006, approved 1 February 2017). For the biodistribution experiment, 6 week-old C57BL/6 female mice obtained from Charles River Laboratories (Wilmington, MA, USA) were injected subcutaneously with 5 × 10^6^ B16-F10 murine melanoma cells in Matrigel (Corning, Corning, NY, USA) into the right flank, or were not given tumor cells. The radioactivity was administered on Day 8 post tumor cells injection, when the tumors reached 0.7–1.0 cm in diameter.

### 2.5. Biodistribution of Antibody-CHXA” Conjugate in Tumor-Bearing Mice

For the biodistribution of melanin binding antibody (Ab1) B16-F10 tumor-bearing C57BL/6 mice were injected intravenously (IV) via the tail vein with 30 µCi antibody-CHXA”—^111^In in 100 µL saline. The same activity of the radiolabeled non-specific antibody (Ab2) was injected into healthy C57BL/6 mice. At the predetermined time points of 4 and 24 h groups of 4–5 mice were humanely sacrificed, their tumors and major organs removed, blotted from blood, weighed and counted for radioactivity in a gamma counter. The standard was prepared by diluting 10 µL (1/10 of the injected dose) of the respective radiolabeled antibody with 2 mL 0.15 M ammonium acetate buffer and counted in a gamma counter at the same time as the organ/tissues were counted. Percentage of injected dose per gram (ID/g) organ/tissue was calculated by dividing the CPM in an organ by its weight in grams and by the CPM of the standard, followed by multiplying the resulting number by 10.

## 3. Results

### 3.1. Radiolabeling of the Antibodies

Two IgG isotype antibodies, Ab1 to melanin and irrelevant isotype matching control Ab2, were conjugated to the bifunctional chelating agent CHXA” to enable subsequent radiolabeling with ^111^In. The concentration of the conjugated antibodies was 16 mg/mL as per Bradford assay which constitutes the 80% recovery of the antibodies after buffer exchange and subsequent purification on the Amicon microconcentrators. The iTLC analysis of the ^111^In-labeled antibody conjugates revealed a radiolabeling yield of >92%, which allowed for the use of the radiolabeled antibody conjugates in the biodistribution experiment without further purification.

### 3.2. Biodistribution of the Radiolabeled Antibodies in Melanoma Tumor Bearing Mice

The raw data and the calculated % ID/g for a 4 h time point for the ^111^In-Ab1 is shown in Table 1. The complete set of data for both ^111^In-Ab1 and ^111^In-Ab2 and 4 and 24 h time points is presented in Supplementary Table S1 in the form of an Excel file. The columns in Table 1 include the following: rack number (for record keeping purposes), tube number, Ab name, organ/tissue, empty tube weight into which that organ was subsequently placed, weight of the tube with the organ, calculated weight of the organ, the CPM in the organ obtained by counting it on the gamma counter, and finally, the % ID/g which is calculated using a formula described in the Methods section, in footnotes to Table 1 and incorporated into the Excel file in Supplementary Table S1.

To take into consideration the radioactive decay of ^111^In during the experiment, the standards (in our case 1/10 of the injected dose) were counted simultaneously with the organs. Each standard was counted two times, at the beginning and end, and the mean of these two measurements was used in the calculations of the % ID/g. The mean of the two readings allow us to account for the minor decay of ^111^In that occurs during counting of multiple samples in a gamma counter. Table 2 shows the values in CPM for the ^111^In-Ab1 and ^111^In-Ab2 standards for 4 and 24 h time points.

**Figure 1:**
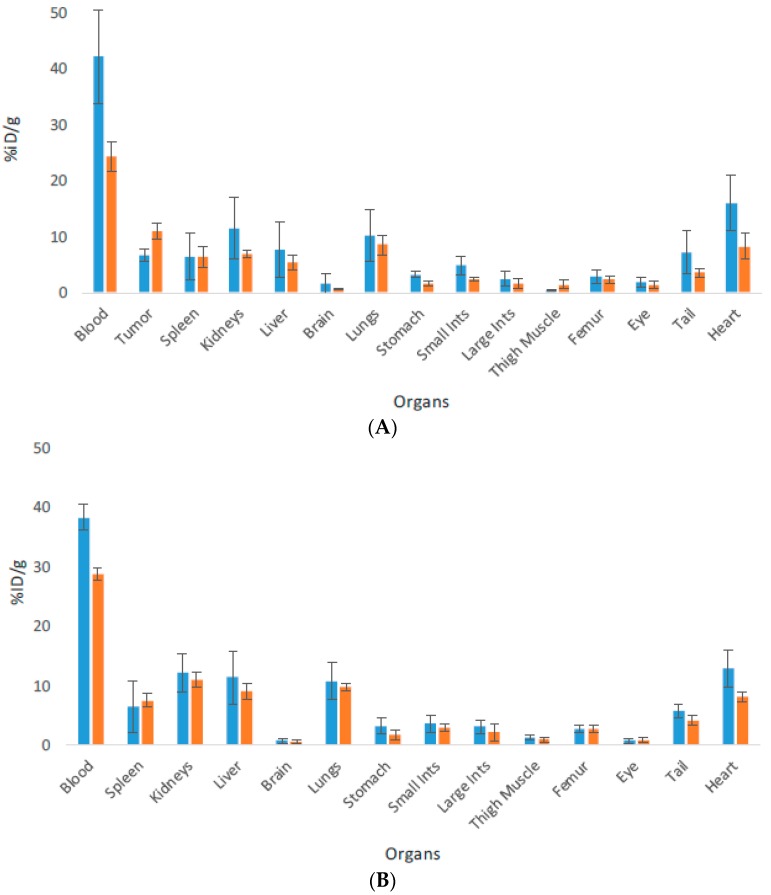
Biodistribution of ^111^In-labeled antibodies Ab1 to melanin and isotype matching irrelevant control Ab2 in C57BL6 mice at 4 and 24 h after IV administration. (**A**) Ab1 antibody in B16F10 melanoma bearing mice; (**B**) Ab2 in healthy mice. Blue and orange bars represent 4 and 24 h time points, respectively. The error bars represent the SEM of sample groups of 4–5 mice.

The process of biodistribution is shown in Supplementary Video S1. The biodistribution was then used to construct biodistribution plots which are presented in Figure 1. The organs and tissues are shown on the *X* axis while the % ID/g for the respective organ/tissues is shown on the *Y* axis. The biodistribution patterns for both antibodies are typical for IgG biodistribution in mice. The clearance from the blood has begun by 24 h, although antibodies are still in circulation at this time point. The antibodies have started to clear from blood rich organs such as heart and lungs. The organs where the antibodies are processed such as spleen, liver and kidneys show very similar uptake for both antibodies, which starts to decrease by 24 h. No penetration of the antibodies in the brain and into the melanized tissues such as eyes is observed. There is very insignificant uptake into stomach, small and large intestines, femur and muscle. The melanin binding antibody Ab1 accumulates in the tumor in B16F10 tumor bearing mice and its uptake increases with time, indicating that it is specific to melanin present in the tumors.

## 4. Discussion

With the increasing pace of drug discovery, in particular antibody-based drugs, faster and more cost-effective methods to assess PK parameters are required. Utilizing commercially available linkers and radioisotopes, we are able to improve upon traditional pharmacokinetic studies by reducing time and cost investments while increasing reproducibility. Here we describe the radiolabeling and biodistribution of two IgG isotype antibodies in tumor bearing versus healthy mice. Ab1 is specific for the pigment melanin, while Ab2 is an isotype matching control. The radiolabeling procedure, which involves using commercially available bifunctional chelating agent CHXA” and radionuclide ^111^In, is straightforward and does not require post-labeling purification. Mice used in the biodistribution could be either healthy if only PK data is being collected, or could be a model of a disease of interest. For this study we used murine melanoma tumor bearing mice for the melanin-binding antibody and healthy mice for isotype matching controls.

In this study, we collected the data at two time points of 4 and 24 h. These time points were chosen to obtain initial evidence of specific targeting. While these time points do not constitute a complete PK study, they do provide sufficient evidence that this method for assessing PK parameters is sensitive enough to detect the changes in targeting and accumulation over time. Depending on the nature of the research, much earlier or much later time points could be collected for more extensive longitudinal studies assessing adsorption, distribution, metabolism, and excretion. For example, when the ^111^In radiolabel is used for labeling antibodies the pharmacokinetics in mice could be followed for up to 7 days, as the physical half-life is 2.8 days. If much later time points are desirable, longer lived radionuclides such ^177^Lutetium, which also forms stable complexes with CHXA” bifunctional chelating agent [6] and has a 6.7-day physical half-life, could be used in place of ^111^In. Collection of data from at least 4–5 time points would enable the modeling of blood clearance of the antibodies using biphasic models, as well as allowing radiation dosimetry calculations if an antibody-based imaging or radioimmunotherapeutic agent is being developed. This method is sensitive as we were detecting ^111^In at the 150–1200 nCi level, which correlates to 75–600 ng amounts of Ab, based on the 2 µCi/µg specific activity. The use of metabolic cages during the biodistribution experiments would enable collection of urine and feces, which could be counted in a gamma counter in the same way as the organs, and would provide a wealth of information on the metabolic fate of the antibody. Finally, if non-invasive imaging equipment such as microSPECT (micro Single Photon Emission Computed Tomography) or microPET (micro Positron Emission Tomography) is available [7] and is equipped with the software allowing quantification of the radiolabeled antibody uptake in the organs/tissue, one group of 4–5 mice per antibody could be followed longitudinally without the need to sacrifice animals at every time point, greatly reducing the number of animals used in the experiment.

## 5. Conclusions

In conclusion, here we describe a relatively simple and efficient way to study the pharmacokinetics of antibodies by radiolabeling them with commercially available radionuclides and performing biodistribution, either in healthy mice or in disease models. We, as well as other groups have successfully used this technique for preclinical development of antibodies for therapy or for imaging of cancer, infection, and neurodegenerative diseases [8,9,10,11,12,13,14,15,16,17,18,19,20].

## Figures and Tables

**Table 1:** Example of the raw data and the calculated uptake of the ^111^In-Ab1 into the organs/tissues at 4 h post administration to mice.

Rack #	Tube #	Antibody	Time Point, h	Organ	Tube Weight, g	Tube + Organ, g	Organ Weight, g	Organ CPM *	Formula **
7	1	Ab1	4	Blood	2.6051	2.6621	0.057	1,045,501.78	51.1224844
7	2	Ab1	4	Tumor	2.849	2.9611	0.1121	61,454.48	1.5279529
7	3	Ab1	4	Spleen	2.7385	2.8142	0.0757	52,319.71	1.92633351
7	4	Ab1	4	Kidneys	2.8507	3.1053	0.2546	538,036.52	5.89000124
7	5	Ab1	4	Liver	2.7795	3.2932	0.5137	392,858.5	2.13151611
7	6	Ab1	4	Brain	2.7548	3.0212	0.2664	66,697.57	0.69781102
7	7	Ab1	4	Lungs	2.7711	2.9214	0.1503	832,976.76	15.4467079
7	8	Ab1	4	Stomach	2.8467	3.0603	0.2136	271,074.71	3.53711992
7	9	Ab1	4	Small Ints	2.7852	3.1185	0.3333	462,766.45	3.86980055
7	10	Ab1	4	Large Ints	2.759	3.1762	0.4172	111,965.48	0.74800048
7	11	Ab1	4	Thigh Muscle	2.7371	2.8462	0.1091	9180.22	0.23452566
7	12	Ab1	4	Femur	2.7765	2.9106	0.1341	145,455.67	3.02317932
7	13	Ab1	4	Eye	2.6675	2.6907	0.0232	23,482.75	2.82112935
7	14	Ab1	4	Tail	2.8006	2.843	0.0424	104,294.99	6.85582338
7	15	Ab1	4	Heart	2.731	2.8187	0.0877	267,839.25	8.51209882

#—number; * CPM—counts per minute. ** Formula—Percentage of injected dose per gram (ID/g) organ/tissue was calculated by dividing the CPM (counts per minute) in an organ by its weight in grams and by the CPM of the standard, followed by multiplying the resulting number by 10.

**Table 2:** CPM for the ^111^In-Ab1 and ^111^In-Ab2 standards for 4 and 24 h time points.

Standard	Time Point	Reading 1	Reading 2	Average
Ab1	4	3,619,311	3,556,450	3,587,880.5
Ab2	4	3,391,460	3,328,188	3,359,824
Ab1	24	3,371,690	3,238,291	3,304,990.5
Ab2	24	3,148,796	3,022,212	3,085,504

## Supplementary Materials

The following are available online at http://www.mdpi.com/1999-4923/10/4/262/s1, Table S1: Raw data for biodistribution; Video S1: Evaluating Pharmacokinetics of an Antibody by Radiolabeling and Performing Biodistribution in Mice.
