# Supplementary material for: Biodistribution of a Radiolabeled Antibody in Mice as an Approach to Evaluating Antibody Pharmacokinetics
Source: Pharmaceutics. 2018 Dec 5;10(4):262. doi: 10.3390/pharmaceutics10040262 (PMC6320949; doi:10.3390/pharmaceutics10040262)
Supplement: Supplementary file 1 [file pharmaceutics-10-00262-s001.zip › pharmaceutics-388907-supplementary formatted.pdf]

pharmaceutics

Supplementary Materials: Biodistribution of a Radiolabeled Antibody in Mice as an Approach to Evaluating Antibody Pharmacokinetics

Kevin J. H. Allen, Rubin Jiao, Mackenzie E. Malo, Connor Frank and Ekaterina Dadachova

Table S1. Raw data for biodistribution.

Rack #	Tube #	Antibody	Time Point, h	Organ	Tube Weight, g	Tube + Organ, g	Organ Weight, g	Organ CPM *	Formula **
8	1	Ab1	4	Blood	2.8457	2.9946	0.1489	1720311.58	32.2013771
8	2	Ab1	4	Tumor	2.8397	3.0563	0.2166	462052.74	5.94559233
8	3	Ab1	4	Spleen	2.7629	2.8408	0.0779	245315.74	8.77707859
8	4	Ab1	4	Kidneys	2.7721	3.042	0.2699	1092003.07	11.2767254
8	5	Ab1	4	Liver	2.7744	3.2686	0.4942	1491414.84	8.41119593
8	6	Ab1	4	Brain	2.7623	2.9385	0.1762	19933.6	0.31531299
8	7	Ab1	4	Lungs	2.773	2.9306	0.1576	834163.38	14.752205
8	8	Ab1	4	Stomach	2.7621	2.9241	0.162	179411.04	3.08671249
8	9	Ab1	4	Small Ints	2.7537	2.9922	0.2385	269388.98	3.14813591
8	10	Ab1	4	Large Ints	2.7753	3.0829	0.3076	400930.43	3.63282702
8	11	Ab1	4	Thigh Muscle	2.7033	3.8252	1.1219	11751.91	0.02919553
8	12	Ab1	4	Femur	2.7858	2.9228	0.137	145833.87	2.9668792
8	13	Ab1	4	Eye	2.7602	2.782	0.0218	19186.7	2.45304673
8	14	Ab1	4	Tail	2.8188	2.8599	0.0411	44183.38	2.99625757
8	15	Ab1	4	Heart	2.7691	2.9033	0.1342	698149.26	14.4996593
9	1	Ab1	4	Blood	2.6813	2.6941	0.0128	9705.51	2.1133451
9	2	Ab1	4	Tumor	2.7262	2.8605	0.1343	277988.33	5.76915998
9	3	Ab1	4	Spleen	2.7719	2.8242	0.0523	86804.1	4.62594623
9	4	Ab1	4	Kidneys	2.7294	2.9722	0.2428	1575741.91	18.0883284
9	5	Ab1	4	Liver	2.7588	3.2321	0.4733	2339304.57	13.7756562
9	6	Ab1	4	Brain	2.7626	2.885	0.1224	214819.48	4.89163759
9	7	Ab1	4	Lungs	2.7857	2.8778	0.0921	193369.86	5.85182302
9	8	Ab1	4	Stomach	2.6094	2.8657	0.2563	261158.41	2.83999411
9	9	Ab1	4	Small Ints	2.7508	3.0049	0.2541	643969.8	7.06354744

9	10	Ab1	4	Large Ints	2.6111	2.8681	0.257	141764.44	1.53743305
9	11	Ab1	4	Thigh Muscle	2.7631	2.8722	0.1091	8449.56	0.2158596
9	12	Ab1	4	Femur	2.7867	2.9531	0.1664	240761.09	4.03269159
9	13	Ab1	4	Eye	2.7778	2.796	0.0182	11643.78	1.78313668
9	14	Ab1	4	Tail	2.765	2.8077	0.0427	173501.95	11.3250079
9	15	Ab1	4	Heart	2.7681	2.9113	0.1432	916879.87	17.8456122
10	1	Ab1	4	Blood	2.7712	2.8889	0.1177	1652230.31	39.1251623
10	2	Ab1	4	Tumor	2.6881	2.9061	0.218	654710.61	8.37056775
10	3	Ab1	4	Spleen	2.7598	2.836	0.0762	336685.75	12.3149257
10	4	Ab1	4	Kidneys	2.7662	3.0429	0.2767	1597861.86	16.0950415
10	5	Ab1	4	Liver	2.7645	3.2799	0.5154	635678.47	3.43759824
10	6	Ab1	4	Brain	2.8482	2.988	0.1398	35344.72	0.70465965
10	7	Ab1	4	Lungs	2.7985	2.9058	0.1073	339530.17	8.81943328
10	8	Ab1	4	Stomach	2.7428	3.1127	0.3699	375157.64	2.82677664
10	9	Ab1	4	Small Ints	2.7607	3.0165	0.2558	426901.44	4.65145806
10	10	Ab1	4	Large Ints	2.7793	3.1503	0.371	337739.67	2.53729051
10	11	Ab1	4	Thigh Muscle	2.7404	3.0232	0.2828	63691.81	0.62772036
10	12	Ab1	4	Femur	2.8415	3.0684	0.2269	81110.51	0.99633335
10	13	Ab1	4	Eye	2.7654	2.7827	0.0173	6882.92	1.10889046
10	14	Ab1	4	Tail	2.8346	2.9054	0.0708	106746.89	4.20227049
10	15	Ab1	4	Heart	2.7772	2.946	0.1688	1293762.8	21.3621149
11	1	Ab1	4	Blood	2.7461	2.8509	0.1048	1741150.05	46.3059659
11	2	Ab1	4	Tumor	2.7096	2.7626	0.053	122157.47	6.42400973
11	3	Ab1	4	Spleen	2.7383	2.8183	0.08	121424.54	4.23037152
11	4	Ab1	4	Kidneys	2.7379	2.984	0.2461	574366.57	6.50488422
11	5	Ab1	4	Liver	2.7434	3.0964	0.353	1404352.99	11.0882653
11	6	Ab1	4	Brain	2.7656	2.905	0.1394	46899.7	0.93771167
11	7	Ab1	4	Lungs	2.7667	2.8234	0.0567	122652.01	6.02911602
11	8	Ab1	4	Stomach	2.8529	3.0828	0.2299	327514.85	3.97058096
11	9	Ab1	4	Small Ints	2.7407	2.9066	0.1659	373442.38	6.2739239
11	10	Ab1	4	Large Ints	2.7705	3.087	0.3165	414186.58	3.64740794
11	11	Ab1	4	Thigh Muscle	2.7016	2.7619	0.0603	7751.28	0.35827635
11	12	Ab1	4	Femur	2.7515	2.9726	0.2211	243359.6	3.06776276
11	13	Ab1	4	Eye	2.8001	2.8188	0.0187	7145.88	1.06506507

11	14	Ab1	4	Tail	2.7368	2.7852	0.0484	186981.86	10.7675313
11	15	Ab1	4	Heart	2.8461	2.9668	0.1207	781534.92	18.0469223
13	1	Ab2	4	Blood	2.8389	2.9962	0.1573	1962515.31	37.1336639
13	2	Ab2	4	Spleen	2.8333	2.9479	0.1146	97137.36	2.52281352
13	3	Ab2	4	Kidneys	2.6082	2.8445	0.2363	247373.75	3.11582719
13	4	Ab2	4	Liver	2.7043	3.1426	0.4383	808791.73	5.49222984
13	5	Ab2	4	Brain	2.7693	2.9749	0.2056	22166.3	0.32088807
13	6	Ab2	4	Lungs	2.7701	2.8819	0.1118	388923.05	10.353933
13	7	Ab2	4	Stomach	2.743	2.9262	0.1832	212189.16	3.4473168
13	8	Ab2	4	Small Ints	2.7586	2.9812	0.2226	386598.19	5.16913802
13	9	Ab2	4	Large Ints	2.7382	2.9447	0.2065	71560.56	1.0314238
13	10	Ab2	4	Thigh Muscle	2.6655	2.8624	0.1969	87470.26	1.32220311
13	11	Ab2	4	Femur	2.8514	3.0298	0.1784	145219.61	2.42277949
13	12	Ab2	4	Eye	2.7337	2.7516	0.0179	3155.75	0.52472654
13	13	Ab2	4	Tail	2.7836	2.8681	0.0845	124142.43	4.37267304
13	14	Ab2	4	Heart	2.6852	2.765	0.0798	268607.49	10.0184077
15	1	Ab2	4	Blood	2.7556	3.066	0.3104	3939783.03	37.777574
15	2	Ab2	4	Spleen	2.7829	2.8644	0.0815	151485.49	5.5321876
15	3	Ab2	4	Kidneys	2.767	3.0298	0.2628	971600.27	11.0038773
15	4	Ab2	4	Liver	2.6924	3.2122	0.5198	2294908.06	13.1405181
15	5	Ab2	4	Brain	2.7394	2.8282	0.0888	19347.8	0.64848823
15	6	Ab2	4	Lungs	2.777	2.9373	0.1603	389545.11	7.23282082
15	7	Ab2	4	Stomach	2.7632	3.038	0.2748	298882.25	3.23718014
15	8	Ab2	4	Small Ints	2.7329	2.9761	0.2432	208534.35	2.55209892
15	9	Ab2	4	Large Ints	2.6962	3.1571	0.4609	538152.09	3.47521594
15	10	Ab2	4	Thigh Muscle	2.7587	3.0731	0.3144	139078.68	1.31662317
15	11	Ab2	4	Femur	2.7569	2.9493	0.1924	215947.56	3.34061714
15	12	Ab2	4	Eye	2.7812	2.8027	0.0215	10651.82	1.47458167
15	13	Ab2	4	Tail	2.8517	2.9861	0.1344	265821.18	5.8867255
15	14	Ab2	4	Heart	2.7705	2.8722	0.1017	482478.25	14.1201809
18	1	Ab2	4	Blood	2.6154	2.791	0.1756	2449112.07	41.5114228
18	2	Ab2	4	Spleen	2.7703	2.8375	0.0672	248515.99	11.0069891
18	3	Ab2	4	Kidneys	2.7735	3.0299	0.2564	1049059.56	12.1777091
18	4	Ab2	4	Liver	2.8005	3.2948	0.4943	1551048.77	9.33938578

18	5	Ab2	4	Brain	2.7762	2.947	0.1708	34858.45	0.60744041
18	6	Ab2	4	Lungs	2.751	2.8936	0.1426	751196.96	15.6789786
18	7	Ab2	4	Stomach	2.6179	2.9551	0.3372	283424.43	2.50168825
18	8	Ab2	4	Small Ints	2.8531	3.0793	0.2262	125586.78	1.65247462
18	9	Ab2	4	Large Ints	2.78	3.167	0.387	448103.9	3.44628532
18	10	Ab2	4	Thigh Muscle	2.7612	2.8736	0.1124	34784.07	0.92108043
18	11	Ab2	4	Femur	2.7721	2.9787	0.2066	124158.48	1.7886672
18	12	Ab2	4	Eye	2.7882	2.8105	0.0223	7106.98	0.94855759
18	13	Ab2	4	Tail	2.8528	2.93	0.0772	165852.37	6.39422411
18	14	Ab2	4	Heart	2.7636	2.8948	0.1312	769892.99	17.4654589
19	1	Ab2	4	Blood	2.8013	2.8916	0.0903	1100948.64	36.287979
19	2	Ab2	4	Spleen	2.7828	2.8512	0.0684	262211.64	11.4098343
19	3	Ab2	4	Kidneys	2.6636	2.9118	0.2482	1387398.14	16.6372983
19	4	Ab2	4	Liver	2.7548	3.1849	0.4301	2562251.62	17.7311052
19	5	Ab2	4	Brain	2.7343	3.0531	0.3188	171994.53	1.60575689
19	6	Ab2	4	Lungs	2.7718	2.8158	0.044	152651.75	10.326011
19	7	Ab2	4	Stomach	2.6739	2.9299	0.256	476700.54	5.54228878
19	8	Ab2	4	Small Ints	2.7659	3.0643	0.2984	429726.07	4.28623872
19	9	Ab2	4	Large Ints	2.7718	3.1586	0.3868	548371.53	4.21960605
19	10	Ab2	4	Thigh Muscle	2.8372	2.9392	0.102	62418.09	1.82135153
19	11	Ab2	4	Femur	2.8528	3.0827	0.2299	253274.25	3.27895562
19	12	Ab2	4	Eye	2.7841	2.804	0.0199	2293.76	0.34306655
19	13	Ab2	4	Tail	2.7739	2.8511	0.0772	186262.69	7.18111766
19	14	Ab2	4	Heart	2.6655	2.7839	0.1184	541299.15	13.6072109
25	1	Ab1	24	Blood	2.7833	3.1102	0.3269	2667984.56	24.6943828
25	2	Ab1	24	Tumor	2.7264	2.9234	0.197	804870.21	12.3620191
25	3	Ab1	24	Spleen	2.8241	2.8983	0.0742	104225.93	4.25012456
25	4	Ab1	24	Kidneys	2.8461	3.094	0.2479	561696.98	6.85575591
25	5	Ab1	24	Liver	2.7397	3.1562	0.4165	956304.37	6.94721787
25	6	Ab1	24	Brain	2.6976	2.9929	0.2953	59222.27	0.60680813
25	7	Ab1	24	Lungs	2.7268	2.854	0.1272	364953.51	8.68120912
25	8	Ab1	24	Stomach	2.7983	3.3842	0.5859	210511.24	1.08713024
25	9	Ab1	24	Small Ints	2.7021	3.0406	0.3385	255352.73	2.28250437
25	10	Ab1	24	Large Ints	2.7198	3.0801	0.3603	189094.7	1.58797945

25	11	Ab1	24	Thigh Muscle	2.7565	2.9417	0.1852	117558.47	1.92062566
25	12	Ab1	24	Femur	2.7538	2.9977	0.2439	195292.2	2.42271809
25	13	Ab1	24	Eye	2.6418	2.6631	0.0213	7485.04	1.06327183
25	14	Ab1	24	Tail	2.7164	2.8075	0.0911	112230.65	3.72754492
25	15	Ab1	24	Heart	2.7647	2.8649	0.1002	231412.21	6.98792657
26	1	Ab1	24	Blood	2.7961	2.9433	0.1472	1001921.5	20.5947095
26	2	Ab1	24	Tumor	2.8534	3.8493	0.9959	3106384.56	9.43776743
26	3	Ab1	24	Spleen	2.7377	2.8095	0.0718	39824.8	1.67825884
26	4	Ab1	24	Kidneys	2.7311	2.9523	0.2212	568667.2	7.77862511
26	5	Ab1	24	Liver	2.8346	3.4945	0.6599	873692.67	4.0059945
26	6	Ab1	24	Brain	2.7631	2.9537	0.1906	42648.87	0.67704014
26	7	Ab1	24	Lungs	2.6622	2.7273	0.0651	234437.15	10.8962041
26	8	Ab1	24	Stomach	2.7621	3.0255	0.2634	144291.31	1.65750249
26	9	Ab1	24	Small Ints	2.7627	2.9861	0.2234	139113.96	1.88415805
26	10	Ab1	24	Large Ints	2.7501	3.0806	0.3305	287251.72	2.62978933
26	11	Ab1	24	Thigh Muscle	2.7495	3.0651	0.3156	53105.88	0.50913787
26	12	Ab1	24	Femur	2.7652	2.9477	0.1825	99597	1.6512513
26	13	Ab1	24	Eye	2.6997	2.7218	0.0221	8217.12	1.12501196
26	14	Ab1	24	Tail	2.756	2.9126	0.1566	211113.24	4.07899805
26	15	Ab1	24	Heart	2.6543	2.7538	0.0995	383384.87	11.6584731
28	1	Ab1	24	Blood	2.768	2.9688	0.2008	1700121	25.6180405
28	2	Ab1	24	Tumor	2.731	3.3401	0.6091	2239732.88	11.1259579
28	3	Ab1	24	Spleen	2.7734	2.8628	0.0894	216915.6	7.3414704
28	4	Ab1	24	Kidneys	2.6692	2.9858	0.3166	674752.17	6.44856622
28	5	Ab1	24	Liver	2.767	3.2555	0.4885	972937.24	6.02629022
28	6	Ab1	24	Brain	2.7617	3.1678	0.4061	63402.52	0.47239285
28	7	Ab1	24	Lungs	2.7348	2.8113	0.0765	174241.18	6.89158557
28	8	Ab1	24	Stomach	2.7014	2.9441	0.2427	135524.48	1.6895761
28	9	Ab1	24	Small Ints	2.7586	3.1486	0.39	294807.65	2.28719886
28	10	Ab1	24	Large Ints	2.7983	3.2077	0.4094	200028.16	1.47833577
28	11	Ab1	24	Thigh Muscle	2.5991	2.8194	0.2203	107991.19	1.48321331
28	12	Ab1	24	Femur	2.7833	3.03	0.2467	210656.36	2.5836591
28	13	Ab1	24	Eye	2.6064	2.6273	0.0209	15305.55	2.2158096
28	14	Ab1	24	Tail	2.7244	2.7644	0.04	32425.59	2.45277483

28	15	Ab1	24	Heart	2.7575	2.877	0.1195	283379.79	7.17514618
29	1	Ab1	24	Blood	2.6153	2.6755	0.0602	525087.2	26.3915395
29	2	Ab1	24	Tumor			0	0	0
29	3	Ab1	24	Spleen	2.7707	2.8232	0.0525	132101.86	7.61341343
29	4	Ab1	24	Kidneys	2.7248	2.9628	0.238	503723.87	6.40391205
29	5	Ab1	24	Liver	2.7555	3.3497	0.5942	868078.21	4.42034322
29	6	Ab1	24	Brain	2.7992	3.0581	0.2589	37190.7	0.4346424
29	7	Ab1	24	Lungs	2.7671	2.8242	0.0571	148302.59	7.85854984
29	8	Ab1	24	Stomach	2.7723	3.0085	0.2362	146005.43	1.87033284
29	9	Ab1	24	Small Ints	2.7843	3.179	0.3947	356257.97	2.73103488
29	10	Ab1	24	Large Ints	2.7026	3.1195	0.4169	62547.54	0.45395011
29	11	Ab1	24	Thigh Muscle	2.8055	3.1264	0.3209	211273.08	1.99206833
29	12	Ab1	24	Femur	2.7973	3.0003	0.203	210817.39	3.14224598
29	13	Ab1	24	Eye	2.7473	2.7677	0.0204	7982.25	1.18392705
29	14	Ab1	24	Tail	2.7606	2.8114	0.0508	64800.34	3.85960941
29	15	Ab1	24	Heart	2.7773	2.8642	0.0869	207354.31	7.21976522
33	1	Ab2	24	Blood	2.7429	3.0671	0.3242	2965223.97	29.6427423
33	2	Ab2	24	Spleen	2.7701	2.8644	0.0943	251900.29	8.65746732
33	3	Ab2	24	Kidneys	2.7715	3.0555	0.284	871413.87	9.94443273
33	4	Ab2	24	Liver	2.7065	3.3076	0.6011	2030151.41	10.9460036
33	5	Ab2	24	Brain	2.8361	3.2441	0.408	120398.71	0.95639117
33	6	Ab2	24	Lungs	2.7323	2.8604	0.1281	418977.81	10.600242
33	7	Ab2	24	Stomach	2.7308	3.2764	0.5456	203725.17	1.21016396
33	8	Ab2	24	Small Ints	2.7615	3.1245	0.363	277203.86	2.4749506
33	9	Ab2	24	Large Ints	2.7866	3.3057	0.5191	263363.55	1.64429041
33	10	Ab2	24	Thigh Muscle	2.6623	2.9763	0.314	108495.1	1.11983584
33	11	Ab2	24	Femur	2.768	2.9658	0.1978	215310.8	3.52787683
33	12	Ab2	24	Eye	2.6642	2.6818	0.0176	6966.64	1.28287573
33	13	Ab2	24	Tail	2.6154	2.7112	0.0958	150736.43	5.09948833
33	14	Ab2	24	Heart	2.7675	2.868	0.1005	272419.11	8.7850734
34	1	Ab2	24	Blood	2.7604	3.0391	0.2787	2539213.49	29.5281429
34	2	Ab2	24	Spleen	2.7494	2.8343	0.0849	218875.21	8.35531392
34	3	Ab2	24	Kidneys	2.7562	3.0246	0.2684	898997.18	10.8554968
34	4	Ab2	24	Liver	2.6725	3.2611	0.5886	1487844.99	8.19240314

34	5	Ab2	24	Brain	2.7379	3.0609	0.323	44081.49	0.44231089
34	6	Ab2	24	Lungs	2.6152	2.6897	0.0745	215871.29	9.39101427
34	7	Ab2	24	Stomach	2.6785	3.021	0.3425	162619.85	1.53881661
34	8	Ab2	24	Small Ints	2.758	3.151	0.393	293430.53	2.4198399
34	9	Ab2	24	Large Ints	2.7608	3.2291	0.4683	280542.15	1.94154674
34	10	Ab2	24	Thigh Muscle	2.6821	2.8216	0.1395	65606.93	1.5242261
34	11	Ab2	24	Femur	2.7749	3.0101	0.2352	207757.25	2.86281152
34	12	Ab2	24	Eye	2.7897	2.8083	0.0186	1875.32	0.3267656
34	13	Ab2	24	Tail	2.7803	2.9124	0.1321	154447.15	3.78923
34	14	Ab2	24	Heart	2.8443	2.9474	0.1031	262946.18	8.26574659
35	1	Ab2	24	Blood	2.7382	3.0171	0.2789	2450738.03	28.4788378
35	2	Ab2	24	Spleen	2.6629	2.7422	0.0793	159692.76	6.52658391
35	3	Ab2	24	Kidneys	2.8448	2.9755	0.1307	503873.55	12.4945282
35	4	Ab2	24	Liver	2.774	3.3002	0.5262	1436856.72	8.84986215
35	5	Ab2	24	Brain	2.6124	2.9362	0.3238	27092.16	0.2711694
35	6	Ab2	24	Lungs	2.7988	2.8871	0.0883	261258.07	9.58921149
35	7	Ab2	24	Stomach	2.7121	2.9662	0.2541	107576.47	1.37210236
35	8	Ab2	24	Small Ints	2.6097	2.875	0.2653	294673.36	3.59979286
35	9	Ab2	24	Large Ints	2.7833	3.2434	0.4601	579524.02	4.08218887
35	10	Ab2	24	Thigh Muscle	2.7016	2.8588	0.1572	32663.88	0.67342482
35	11	Ab2	24	Femur	2.7379	2.9516	0.2137	130361.36	1.9770527
35	12	Ab2	24	Eye	2.7349	2.7529	0.018	6302	1.13469667
35	13	Ab2	24	Tail	2.7842	2.8558	0.0716	72888.61	3.29929031
35	14	Ab2	24	Heart	2.8038	2.8881	0.0843	178686.8	6.86971599
36	1	Ab2	24	Blood	2.7694	2.8818	0.1124	946830.23	27.3010714
36	2	Ab2	24	Spleen	2.764	2.8471	0.0831	164774.22	6.42631674
36	3	Ab2	24	Kidneys	2.7662	3.0375	0.2713	308273.11	3.68264399
36	4	Ab2	24	Liver	2.7275	3.3507	0.6232	1648580.83	8.57347108
36	5	Ab2	24	Brain	2.7671	3.0132	0.2461	12748.47	0.16788826
36	6	Ab2	24	Lungs	2.7681	2.8263	0.0582	40337.37	2.24625202
36	7	Ab2	24	Stomach	2.7539	2.8947	0.1408	128860	2.96612438
36	8	Ab2	24	Small Ints	2.7858	3.1275	0.3417	349303.2	3.31307656
36	9	Ab2	24	Large Ints	2.8453	3.3276	0.4823	115879.01	0.77868427
36	10	Ab2	24	Thigh Muscle	2.7863	2.8605	0.0742	10169.64	0.44419694

36	11	Ab2	24	Femur	2.8	2.9928	0.1928	138791.15	2.33307463
36	12	Ab2	24	Eye	2.7655	2.7836	0.0181	3625.82	0.64923444
36	13	Ab2	24	Tail	2.7328	2.8367	0.1039	142646.44	4.44958263
36	14	Ab2	24	Heart	2.7557	2.857	0.1013	270355.06	8.64965814
